# Development of a Low-Cost Airborne Ultrasound Sensor for the Detection of Brick Joints behind a Wall Painting

**DOI:** 10.3390/s120201299

**Published:** 2012-01-31

**Authors:** Fernando-Juan García-Diego, José María Bravo, Juan Pérez-Miralles, Héctor Estrada, Angel Fernández-Navajas

**Affiliations:** 1 Departamento de Física Aplicada, Universidad Politécnica de Valencia, Av. de los Naranjos s/n, Valencia 46022, Spain; E-Mails: jobrapla@fis.upv.es (J.M.B.); afnavajas@fis.upv.es (A.F.-N.); 2 Centro de Tecnologías Físicas, Unidad Asociada ICMM-CSIC/UPV, Universidad Politécnica de Valencia, Av. de los Naranjos s/n, Valencia 46022, Spain; E-Mail: hector.estrada@icmm.csic.es; 3 Servicio de Conservación y Restauración de la Exma, Diputación de Castellón (SCRC), Complejo Socio-Educativo de Penyeta Roja s/n, Castellón 12080, Spain; E-Mail: jperezm@ivcr.es; 4 Instituto Valenciano de Conservación y Restauración de Bienes Culturales (IVC+R), Complejo Socio-Educativo de Penyeta Roja s/n, Castellón 12080, Spain; 5 Instituto de Ciencia de Materiales de Madrid (CSIC), Cantoblanco, Madrid 28049, Spain

**Keywords:** non destructive test, ultrasound, wall painting, brick joints

## Abstract

Non-destructive methods are of great interest for the analysis of cultural heritage. Among the different possible techniques, this paper presents a low cost prototype based on the emission and reception of airborne ultrasound without direct contact with the test specimen. We successfully performed a method test for the detection of brick joints under a XVth century Renaissance fresco of the Metropolitan Cathedral of the city of Valencia (Spain). Both laboratory and *in situ* results are in agreement. Using this prototype system, an early moisture detection system has been installed in the dome that supports the fresco. The result is encouraging and opens interesting prospects for future research.

## 1. Introduction

In recent years there has been an increasing focus on all areas of heritage conservation in an interdisciplinary approach, especially as regard to systems that allow non destructive analysis. This has been especially true for wall paintings because of their particular vulnerability, as they constitute an external layer which is itself the interface between the support and the environment [1].

In 2004, the metropolitan basilica cathedral of St. Mary in Valencia started the restoration of the main chapel and its Renaissance fresco paintings (http://www.frescosdelacatedral.com) which had been hidden for more than 300 years. In the restoration process the presence of efflorescence was observed and certain parts with deterioration issues due to high humidity were also identified [2]. Since the moisture was restricted to specific zones, it was suspected that the problem could be caused by infiltration of rainwater through the roof above the apse. Attempting to prevent this kind of problems in the future, this roof was remodeled and an asphaltic roofing sheet was laid below the tiles. The importance of waterproofing the outside of these kinds of monuments has been discussed in the literature [3].

Taking into account that it is very difficult to ensure a long-term impermeability of the roof over these paintings that must be conserved over centuries, the restoration team decided to implement a monitoring system comprised by relative humidity sensors. These sensors were installed during the restoration process at different points of the vault, some of them inside the paintings and others outside. This control system is rather unique because sensors are rarely inserted into the precious walls supporting frescoes [4,5].

These holes were made in the brick joints because it is in these areas where humidity appears first. To detect the bricks and its joints, we have developed a straightforward and low-cost airborne ultrasound scan.

The diagnosis of wall paintings using techniques related to sound has evolved in recent years. In the traditional manual acoustic technique, the restorer hits the surface with the knuckles and listens for different sounds which could allow the detection of air gaps [6,7]. However, this manual technique requires special skills to determine the impact force that is applied to the painted plaster and to diagnose possible defects [6].

Among the wide variety of methods for non-destructive testing, elasto-acoustic waves provide a good alternative which has been exploited mainly for biomedical and industrial applications [8–11]. Some of the techniques developed in biomedical and industrial areas have been exported for its use in heritage conservation.

Furthermore, an analysis of acoustic reflections using audible frequencies below 1 kHz in a Kundt tube as an assessment of wall painting detachments has been recently proposed to overcome the drawbacks of the traditional empirical method [6,7,12]. The acoustic method consists in launching an appropriate acoustic wave towards the paint and measure the absorbed acoustic energy, which is then correlated with the degree of detachment.

Another method for studying paint detachment is the vibro-acoustic technique based on the application of the Laser Doppler Vibrometry under acoustic excitation [13]. Both the aforementioned methods only give information about superficial vibrations.

Besides the commented audible frequency methods, the use of ultrasound techniques seems better suited to deal with non-destructive testing of hidden layers from any material [6], since it also provides non-contact experiments together with a higher spatial resolution due to the smaller wavelengths involved in the measurements. Several studies deal with ultrasonic non-destructive contact techniques for the assessment of building materials [14–16].

By using air as a coupling medium, in order to avoid contaminations and damages to the object being tested, new problems must be overcome in setting-up a feasible measurement technique, mainly because of the enormous acoustical impedance mismatch between air and the solid materials involved. Some other problems arise if we consider the highly anisotropic acoustic nature of works of art, for example those made of wood. In the past decades, many ways to overcome these problems have been tried and a considerable number of air-coupled applications are found in literature [9,10,17–20].

In this paper we present a prototype of a non-contact ultrasonic scan method which has been developed to locate joints between bricks in a wall behind a Renaissance fresco. We performed a laboratory experiment to test its effectiveness with materials similar to those of the Metropolitan Cathedral of Valencia and performed *in-situ* measurements using the proposed technique. The effectiveness of the prototype has been proved by locating the position of the joints beneath the frescoes, which allowed the installation of a moisture monitoring system in the paintings. The main advantages of the proposed method, which is crucial for its practical implementation, are its considerably low cost and the small dimensions of the ultrasonic transducers.

## 2. Materials and Methods

### 2.1. Acoustical Method

**Figure 1.**
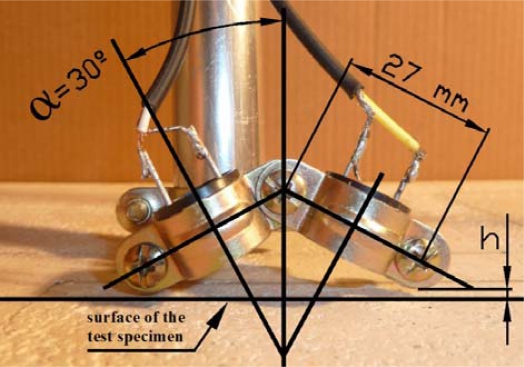
Photograph of the transducers mounting assembly showing the orientation of the transducers. Transmitter and receiver form an angle of α = 30° with the perpendicular of the test specimen. The distance between the bottom of the assembly and the test specimen surface is marked with “h”.

The technique consists in the emission of an ultrasonic signal in an area of study. The reflection of this signal will be picked up by a receiver. The transducers are placed above the surface to be inspected in pitch-catch reflection mode (Figure 1).

A fluid-solid interface exhibits mainly three different types of acoustic behavior: simple transmission-reflection-refraction, leaky Rayleigh waves, and Scholte-Stoneley waves [21]. If the source is placed, as in our case, in the fluid and it has a relatively small size, all of the three aforementioned mechanisms will be mixed. The first mechanism will produce specular reflection into the fluid. Rayleigh waves will propagate through the solid surface leaking energy into the fluid at a certain angle which depends on the Rayleigh wave speed and fluid wave speed. Finally, Scholte-Stoneley waves will propagate right through the fluid-solid interface with a speed slightly lower than that of the fluid. The energy contribution of these three mechanisms depends on the elastic constants of the solids relative to those of the fluid.

When the fluid is air, any conventional solid having low porosity will produce almost the same behavior and the specular reflection will greatly dominates due to the huge impedance mismatch between both media. In fact, considering the density and sound velocity of air, bricks, and gypsum board, given in Table 1 [22], the impedance mismatch *K* can be calculated as:

(1) $$K=\frac{\rho_{i}\;c_{i}}{\rho_{0}\;c_{0}}$$

where *ρ_i_* *c_i_* corresponds to density and sound velocity in the material and *ρ*_0_ *c*_0_ for the air features. Resulting a *K* ≈ 16,626 for brick and *K* ≈ 10,728 for gypsum.

In other words, the reflected signal is practically equal to the incident one, as can be calculated for oblique incidence using a the following relation [23]:

(2) $$R=\frac{\rho_{i}\;c_{i}-\rho_{0}\;c_{0}\;\text{cos}\;\theta_{i}}{\rho_{i}\;c_{i}+\rho_{0}\;c_{0}\;\text{cos}\;\theta_{i}}$$

where *R* is the reflection coefficient and *θ_i_* is the incidence angle. Results for different incident angles are shown in Table 2.

The basis of the proposed method consists in measuring the phase difference produced by the ultrasound reflection at different points of the test specimen under testing. As the signal which drives the emitter is a pure tone, the phase can be easily calculated. The sum of two waves having the same amplitude and frequency but different phase can be written as:

(3) $$x=e^{i\omega t}+e^{i(\omega t+\phi )}$$

where ω is the angular frequency and *ϕ* is the phase shift. Using simple trigonometric relations, one can obtain the phase shift *ϕ* as a function of the RMS amplitude of the real part of x (*x̂*=Re{*x*}) as:

(4) $$\phi =2\;\text{arccos}\left(\frac{{\hat{x}}_{\mathrm{RMS}}}{\sqrt{2}}\right)$$

In practice, this method has been implemented offline as part of the post processing. The reference signal *e^iωt^* has been synthesized and represents the input signal of the emitter. The signal measured by the receiver is normalized in order to use Equation (4). Then, in the acquisition stage, the signal on the receiver is synchronized with the signal generator so that all the points measured have the same phase reference.

### 2.2. Acoustical Apparatus

**Figure 2.**
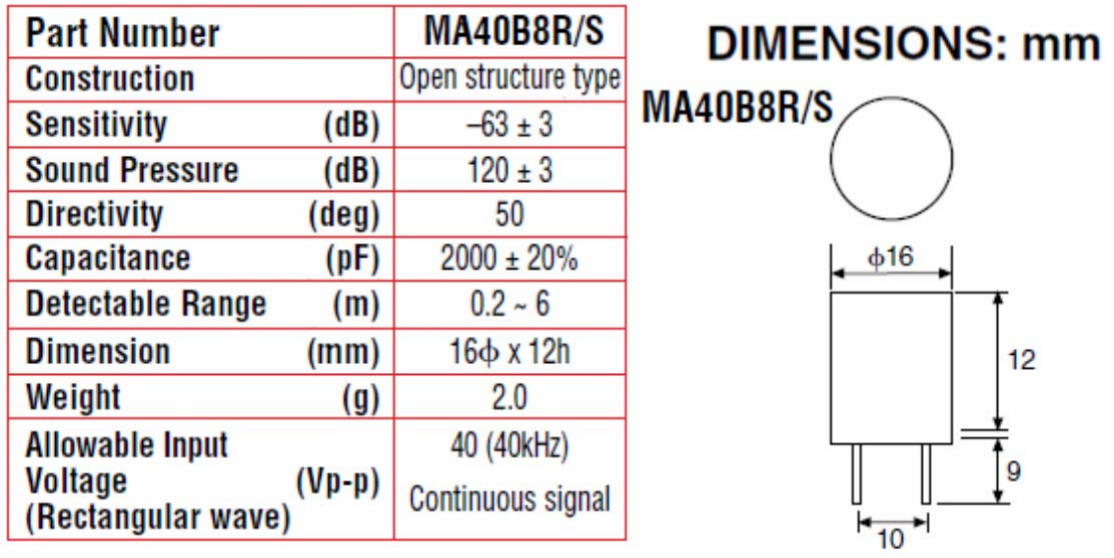
Specifications and dimensions of Murata Manufactoring Co., Ltd. 40 kHz emitter and receiver transducer.

A 40 kHz Murata Manufactoring Co., Ltd (Kyoto, Japan) ultrasonic MA40B8S emitter and a MA40B8R receiver were used. The emitter was driven constantly by a 1.5 volt peak to peak sinusoidal wave. As already mentioned, each of them was mounted forming a 30° angle with the perpendicular axis of the test specimen under study (Figure 1). The transducers cover a bandwidth ranging approximately from 38 kHz to 43 kHz and are 1.6 cm in diameter (Figure 2).

The input signal is generated with an Agilent Technologies (Santa Clara, CA, USA) 33220A signal generator. The received signal was ×10 preamplified by a LM324 AC coupled inverting operational amplifier to obtain more than 200 mV Pk–Pk in the receiver and eventually acquired using a digital PC oscilloscope, Pico Technology (Cambridgeshire, UK) model PicoScope 3224. The data acquisition stage is coordinated with an automated positioning system, described below. In order to increase the signal-to-noise ratio, 50 signals have been averaged. Around five periods of long time domain signals have been post-processed to extract both the amplitude and the phase. Thus, disregarding the time needed to move the sensors along the surface, a single point measurement takes less than 50 ms, including averaging.

### 2.3. X-Y-Z Movement and Data Acquisition

**Figure 3.**
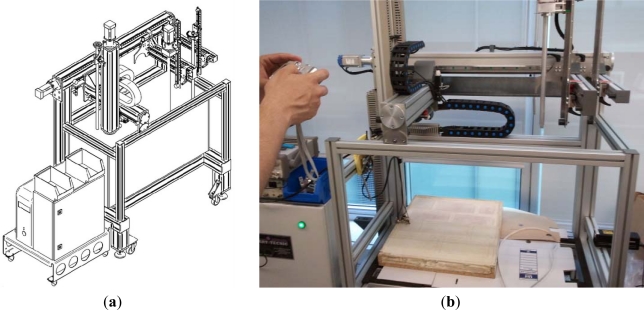
Artitecnic (Ibi, Spain) DS4, X-Y-Z automatic positioning system. (**a**) Schematics; (**b**) Photograph scanning the test specimen.

X-Y-Z positioning was provided by an automated positioning system (Figure 3). Further software and hardware is required to synchronize the position stage and to register the measured data. This equipment is rather standard and will be briefly described as follows. A computer moves the X-Y-Z robot to a previous programmed position, and then the receiver’s signal is digitalized by the digital oscilloscope (Picoscope 3224) and is saved with the position in the PC. The signal generator of the transmitter acts as trigger of the oscilloscope, so all the received data have the same reference. This process is repeated until all programmed points are measured.

The surface measured covers 25.5 × 41.4 cm^2^ in steps of 3 mm in both Cartesian axes X and Y. Thus, one measurement comprises 138 × 85 mesh points, which corresponds to 11,730 measurement positions. The same XY measure was performed at two different heights. The sensor reference line was established at two different heights, 1 and 2 mm, measured from the lower part of our system to the test specimen surface marked as “h” in Figure 1.

### 2.4. 3D Visible Light Scanning

The 3D visible light scanning consists in projecting a specific visible light pattern on the objects surface to extract geometrical information from the deformations of this pattern by triangulation [24–27]. We use a Breuckmann (Meersburg, Germany) model optoTOP-HE scanning system, which has a minimum depth resolution of 2 μm. The test specimen surface was scanned to determine if the phase delay observed in the ultrasound experiments was simply due to height variations of the test specimen surface.

### 2.5. Test Specimen

**Figure 4.**
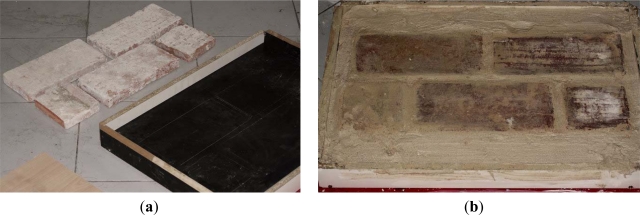
Test specimen preparation: (**a**) positioning of the bricks with a joint of 2 cm; (**b**) Coating with lime and sand mortar.

The test specimen (Figure 4) was built manually with brick clay and mortar of lime and sand. The joints are 2 cm wide and the coating of 7 mm in depth. The test specimen was prepared with materials and techniques similar to those of the Gothic building with the aim that the results could be extrapolated.

Some cuts were made on the surface of the test specimen to locate the corners of the bricks.

### 2.6. Frescoes Measurements

In order to monitor the microclimate that affects the Renaissance frescoes of St. Mary’s Cathedral of Valencia, ceramic tubes were inserted in the joints between the bricks of the vault that supports these frescoes.

Taking advantage of a section of wall where bricks were visible, a template on a transparent plastic sheet was drawn. This plastic was used as a guide of the size of bricks and joints since the arrangement of the bricks beneath the frescoes was known.

**Figure 5.**
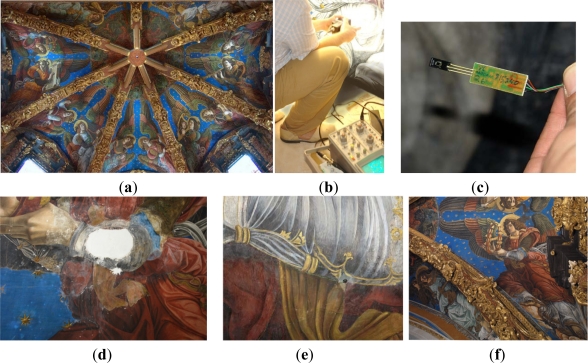
(**a**) Overview of the vault of the Valencia Cathedral; (**b**) Manual scan looking for the sensors insertion points in frescoes; (**c**) Humidity sensor detail; (**d**) Forearm detail of (f) before restoration; (**e**) In the center of the picture, a sensor is inserted by the ultrasound technique in (d); (**f**) Detail of the vault after the restoration.

In areas where we intended to locate the joint a manual scan was performed to mark the points where the phase changes were evident. These points were compared with the template to ensure certainty. Ten points were marked by this technique (Figure 5). Holes of 16 mm in diameter were drilled to a depth of 15 cm and the resulting powder was stored for microscopic analysis to see if its color was red (brick) or white (joint).

## 3. Results and Discussion

### 3.1. Laboratory Measurements

**Figure 6.**
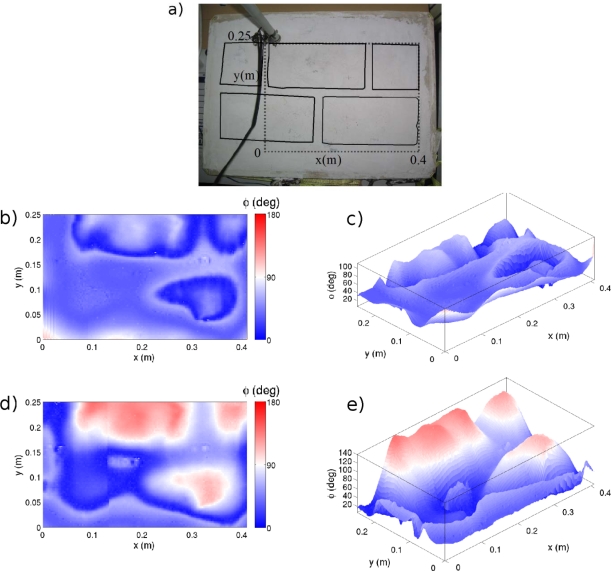
Obtained results: (**a**) Image of the test specimen, where the dashed line marks the scanned area and the continues line depicts the limits of the bricks; (**b**) Two-dimensional representation of phase values measured at h = 1 mm; (**c**) Same results as in (b) represented three-dimensionally; (**d**) Two-dimensional representation of phase values measured at h = 2 mm; (**e**) Same results as in (d) represented three-dimensionally.

An analysis of the phase shift was performed for two different scans at two different heights from the test specimen. The results of the scan are shown in Figure 6. The color scale depicts phase variations from 0 to 180 degrees as a function of the transducers position over the test specimen.

As shown in Figure 6, the offset phase remains constant within the areas composed of the same material. However, at material interfaces (brick-board) phase shows abrupt changes. These abrupt changes allow us to clearly appreciate the limits of the bricks. In general, for the two heights of studied the same results are obtained, indicating the robustness of the phenomenon.

The offset values observed in the two experiences are different. This is because the offset value of a position depends on the distance traveled by the wave between the emission and reception. The height variation produces different values of phase shift, although the pattern is identical.

The bricks in the bottom row are not as well defined as those at the top. This may be due to different causes. First bricks have different thickness due to the handmade fabrication process, *i.e.*, they might be placed at different depths with respect to the test specimen surface. On the other hand the surface of the test specimen is not completely flat, as discussed in the next section and this issue may affect the results.

In addition, we tested another sensor configuration in terms of their orientation. The emission was perpendicular to the test specimen surface and two symmetrical receivers forming an angle to the test specimen were used. However, the results obtained with this configuration did not provide clear information about the position where the bricks are located.

### 3.2. 3D Visual Light Scanning

**Figure 7.**
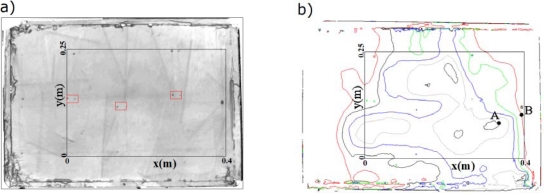
(**a**) Topography grayscale image of the test specimen. The points within the red rectangles show the cuts made to mark the corners of the bricks; (**b**) Contours every 0.25 mm being the lowest in red (0 mm) and the highest in black (1 mm).

As the frequency used was *f* = 40 *kHz*, the wavelength is 8.5 mm (using the speed of sound in air at laboratory constant conditions as *c_o_* = 340 *m*/*s*). This implies that a variation in height between two points produces a delay *φ* given by:

(5) $$\varphi =\frac{\omega }{c_{0}}2\Delta h\;\text{cos}\;\alpha$$

where *ω*= 2*π f* is the angular frequency, Δ*h* is the height variation and *α* = 30° (Figure 1). The topography data (Figure 7(b)) shows that the maximum variation in elevation at the test specimen is 1 mm, which corresponds to a maximum delay of 73.33°.

The maximum change in height variations occurs for distances greater than 6 cm. Figure 7(b) shows that the maximum height gradient exists between points A and B. In the results shown in Figure 6, there are angular delays greater than 73.33° between points located very close to each other. On the other hand, observing the contour lines in Figure 7(b), there is no correspondence with the geometry and brick arrangement of the test specimen.

These two facts indicate that even though the effect of the surface topology is always present in the phase delay, it cannot explain the large and abrupt phase changes we have measured. Furthermore, the cuts made to locate the bricks can be clearly seen in the topographic maps (Figure 7) and correspond to abrupt but small changes of phase gradient in the results shown in Figure 6(b) and, more clearly, by Figure 6(d).

In light of the previous theoretical considerations (see Table 2) our experimental results are very surprising. We seem to be able to detect what lies below the solid coating, which is completely precluded by both the theory and the common sense. However, the nature of the solid components of the test specimen and the way they are bonded together is very important in our case. Besides the academic assumption that the brick-joint interface can be well defined and the continuity of both stress and displacement can be guaranteed, our results may indicate the presence of inhomogeneities containing air in the brick-joint interface.

The analysis of the topography clearly indicates that the measurements are not just the phase difference due to the specular reflection of the incident beam. Although further research is needed to completely understand the physics behind our experimental results, the presence of air inhomogeneities at the interfaces seem to be unavoidable for the materials and the handmade technique employed in fabricating the test specimen and the wall paintings. Then, the efficacy of the proposed airborne ultrasound technique in the assessment of old structures seems very promising.

### 3.3. Frescoes Measurements

We obtained white dust in nine of the ten marked points meaning that all the holes were made in a joint and in the last one, traces of red dust (30% approximately) were found meaning that a small part of a brick was perforated.

## 4. Conclusions

Our results show that abrupt changes in the measured phase are well correlated with the limits of bricks allowing a good location of joints (interface zone). Furthermore, the analysis of the topography clearly indicates that the measurements are not just the phase difference due to the specular reflection of the incident beam on the test specimen surface.

The acoustical method presented here meets the requirements to perform *in situ* non-invasive studies of bricks position supporting mural paintings and simple plasters, and could be extended to other antique artifacts such as mosaics, *etc*. In fact, its simplicity and the low data acquisition time could be put together to obtain real time measurements, which in addition to a position tracking system would enable a fast and versatile non-destructive system.

Although this is a preliminary study and more tests and theoretical simulations must be performed to clearly understand the underlying physical phenomenon, the results are sufficiently promising for its application in other areas within the cultural heritage and even in industrial applications for the detection of defects in structures or surfaces.

## Figures and Tables

**Table 1.** Values of density and velocity for air (*c_o,_* *ρ_o_*), bricks (*c_b,_* *ρ_b_*) and gypsum board (*c_g_*, *ρ_g_*).

*ρ_o_* (*kg*/*m*^3^)	1.21
*ρ_b_* (*kg*/*m*^3^)	1,800
*ρ_g_* (*kg*/*m*^3^)	650
*c_o_* (*m*/*s*)	340
*c_b_* (*m*/*s*)	3,800
*c_g_* (*m*/*s*)	6,790

**Table 2.** Values of the reflection coefficient for three differents angles. The angle of the sensors with the normal of the test specimen surface are 30 degrees.

	Air-brick	Air-gypsum

R (25°)	0.999	0.999
R (30°)	0.999	0.999
R (35°)	1.000	1.000
